# Finerenone as a Third-Line Therapy for Persistent Proteinuria in Diabetic Kidney Transplant Recipients

**DOI:** 10.3390/ijms27114832

**Published:** 2026-05-27

**Authors:** Carmine Secondulfo, Dora Russo, Nicoletta Vecchione, Gianmarco Minelli, Luca Apicella, Candida Iacuzzo, Chiara Crescenzo, Maristella Minco, Anna Sannino, Gennaro Clemente, Antonio Pisani, Massimo Cirillo, Giancarlo Bilancio

**Affiliations:** 1 Department of Medicine, Surgery and Dentistry “Scuola Medica Salernitana”, University of Salerno, 84081 Baronissi, Italymcirillo@unisa.it (M.C.); 2 Department of Public Health, University of Naples “Federico II”, 80131 Naples, Italy; dora.russo94@gmail.com (D.R.); nicolettavecchione@gmail.com (N.V.); gianmarcominelli@gmail.com (G.M.); antonio.pisani13@gmail.com (A.P.); 3 Unit of Nephrology, Dialysis and Transplantation, University Hospital of Salerno “San Giovanni di Dio e Ruggi d’Aragona”, 84131 Salerno, Italy; luca.apicella@sangiovannieruggi.it (L.A.); candida.iacuzzo@sangiovannieruggi.it (C.I.); dottoressacrescenzo@gmail.com (C.C.); maristella.minco@sangiovannieruggi.it (M.M.); anna.sannino94@gmail.com (A.S.); 4 Unit of Diabetology and Nutrition, University Hospital of Salerno “San Giovanni di Dio e Ruggi d’Aragona”, 84131 Salerno, Italy; gennaro.clemente@sangiovannieruggi.it

**Keywords:** finerenone, kidney transplant, proteinuria, albuminuria, diabetes

## Abstract

Proteinuria is a strong predictor of graft failure in kidney transplant recipients (KTRs). While non-steroidal mineralocorticoid receptor antagonists (NS-MRAs), particularly finerenone, have demonstrated renoprotective benefits in chronic kidney disease, KTRs were excluded from pivotal trials. Evidence on finerenone’s safety and antiproteinuric effects in this population remains limited. This retrospective observational study evaluated 13 diabetic KTRs with persistent proteinuria despite optimized renin–angiotensin system blockade and sodium–glucose cotransporter 2 inhibitor therapy. Finerenone (10 mg/day) was added to standard care. Clinical and laboratory parameters, including estimated glomerular filtration rate (eGFR), serum electrolytes, total proteinuria, albuminuria, and their creatinine ratios, were assessed at baseline, 3 months, and 6 months. Safety outcomes focused on hyperkalemia and eGFR. Finerenone was discontinued in one patient due to hyperkalemia. In the remaining 12, 24-h proteinuria and urinary protein-to-creatinine ratio declined at 3 months and stabilized by 6 months. Conversely, no statistically significant changes were observed in albuminuria or the albumin-to-creatinine ratio. No clinically relevant changes occurred in eGFR, blood pressure, body weight, or serum electrolytes. This is the first study assessing finerenone in diabetic KTRs. Finerenone was well tolerated, was associated with an early reduction in proteinuria, and showed no adverse effects on graft function. These findings provide novel insights into the safety and potential role of finerenone in kidney transplant recipients.

## 1. Introduction

Chronic kidney disease (CKD) affects approximately 9.1% of the global population, with a 29.3% increase in prevalence and a 41.5% rise in mortality over recent years [1]. Kidney transplantation remains the preferred modality of renal replacement therapy, offering improved survival, better quality of life, and lower healthcare costs compared to dialysis [2]. Consequently, there is considerable interest in strategies aimed at further prolonging graft survival in kidney transplant recipients (KTRs) [2]. Historically, long-term outcomes after kidney transplantation have remained largely unchanged. However, recent data show modest but measurable improvements in graft survival half-lives, now exceeding 11 years for deceased donors and 19 years for living donors [3]. These gains are attributed not only to an improved understanding of immune-mediated rejection and advances in immunosuppressive therapy but also to better management of comorbidities such as diabetes and hypertension, which significantly influence both patient and graft survival [3,4]. Proteinuria has emerged as a strong predictor of graft failure, and its control may offer a viable strategy to improve long-term outcomes. Notably, while current research primarily addresses its effects on albuminuria, proteinuria in kidney transplant recipients is often linked to chronic tubulointerstitial injury and frequently comprises a substantial component not deriving from glomerular loss [5,6,7,8]. This distinction highlights a potential gap in ongoing studies that may overlook key pathophysiological features relevant to this population.

Two classes of drugs have been recently approved for the prevention of CKD progression and prevention of cardiovascular events in kidney patients: sodium–glucose cotransporter type 2 inhibitors (SGLT2i) and nonsteroidal mineralocorticoid receptor antagonists (NS-MRAs). For both these drug classes, KTRs were excluded from the clinical trials, and there is great interest in their use in these patients.

SGLT2i are currently showing promising effects in KTRs with and without diabetes; mounting evidence suggests a potential benefit for this especially fragile category of patients [8,9,10,11,12,13,14].

NS-MRAs are a novel class of drugs, with finerenone currently the only agent approved for clinical use. Similarly to SGLT2i, NS-MRAs are revolutionizing the therapeutic approach to CKD due to their significant impact on CKD progression and cardiovascular protection [15,16]. Although KTRs were not included in clinical trials on finerenone, current evidences for the use of conventional mineralocorticoid receptor antagonists (MRAs) in this population may support the rationale for further investigation into the use of non-steroidal MRAs in KTRs [17]. MRAs may play a role in the management of selected KTRs due to the high burden of cardiovascular disease; furthermore, MRAs decrease inflammation, endothelial dysfunction and oxidative stress [18,19,20]. However, available studies are few and heterogeneous, preventing definitive conclusions. While concerns about hyperkalemia and declines in GFR are valid, the limited data available have not demonstrated a significantly increased risk of adverse events.

This observational study aims to evaluate the safety, tolerability and effects on proteinuria and glomerular filtration rate of finerenone in a cohort of kidney transplant recipients.

## 2. Results

A total of 13 patients were suitable for enrollment in this analysis. Complete descriptive baseline statistics are available in Table 1. The majority of patients were males and hypertensive. The mean age was 57.7 years, and the mean transplant age was 12.6 years, with a mean eGFR 44 mL/min/1.73 m^2^.

At the 1-month visit, no clinically relevant reduction was found for eGFR in any of the subjects involved (mean serum creatinine 1.95 ± 0.55 mg/dL; mean eGFR 38 ± 12 mL/min/1.73 m^2^). One patient experienced an elevation in serum potassium (6.1 mEq/L), and finerenone was discontinued as a precaution, while no relevant variations in kalemia were observed in any other patient (mean serum potassium 4.8 ± 0.5 mEq/L).

**Figure 1:**
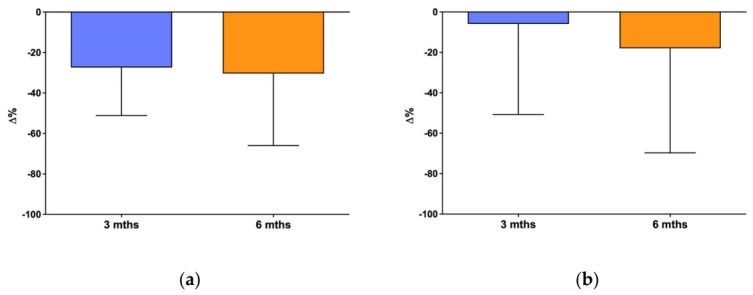
Percentage of reduction in urinary parameters, median (IQR): (**a**) total proteinuria; (**b**) albuminuria.

At 3 months of therapy, median 24 h urinary proteins and median urinary protein-to-creatinine ratio (uPCR) showed a statistically significant reduction. Despite a tendency toward amelioration in median urinary albumin and its ratio to creatinine (uACR), statistical relevance was not achieved. After 6 months of therapy, no further clinically relevant variation in any of the median urinary protein parameters was found, with a substantial stabilization of the above variables (Table 2). The percentage of variation in total proteinuria and albuminuria (27% and 30%, 6% and 18% at 3 and 6 months vs baseline, respectively) confirms this pattern (Figure 1).

No changes in other clinical parameters, such as arterial blood pressure, body weight, eGFR, serum electrolytes, were observed (Table 2).

## 3. Discussion

Proteinuria is a major indicator of allograft injury in kidney transplant recipients, similarly to native chronic kidney disease [21], but with the important difference that proteinuria in KTRs is frequently associated with chronic tubulointerstitial injury [5,6,7,8]. KTRs were excluded from the main trials on finerenone [15,16], and the only available study on finerenone in this particular category of patients, by Kahvecioglu et al., does not address the difference between total proteinuria and albuminuria [22]. Furthermore, in contrast with our study, the cohort described by Kahvecioglu et al. consisted of non-diabetic subjects, and none of the patients were treated with any SGLT2 inhibitor; thus, comparisons between the two studies should be interpreted with considerable caution.

Our work provides novel information on a sample cohort of KTRs that could be used to prompt further investigation and provide clinical guidance.

Regarding eGFR, our data aligns with findings from the analysis by Kahvecioglu et al. on 15 KTRs, which shows a slight increase in serum creatinine in the first month of therapy, but with no significant change in eGFR after three and six months of therapy.

Similarly, in the work by Mortensen et al. on 180 KTRs assuming spironolactone, eGFR decreased after 1 week in the treatment group, but maintained a similar slope over 3 years between treatment and placebo groups [23].

Further confirmation comes from evidence from a 64-patient study by Morales et al. on KTRs treated with spironolactone; a tendency toward lower values of serum creatinine for 3 years after transplantation was observed, but it did not reach statistical significance [24].

In the retrospective study by de Sousa et al. on 140 KTRs treated with spironolactone, eGFR showed an insignificant tendency toward a modest reduction [25], agreeing with previously mentioned findings.

Conversely, in the study by Baskin et al. on 26 pediatric KTRs undergoing treatment with eplerenone, eGFR remained stable over the 3-year observation period in the subgroup treated with MRA, while it declined in the control group [26].

**Figure 2:**
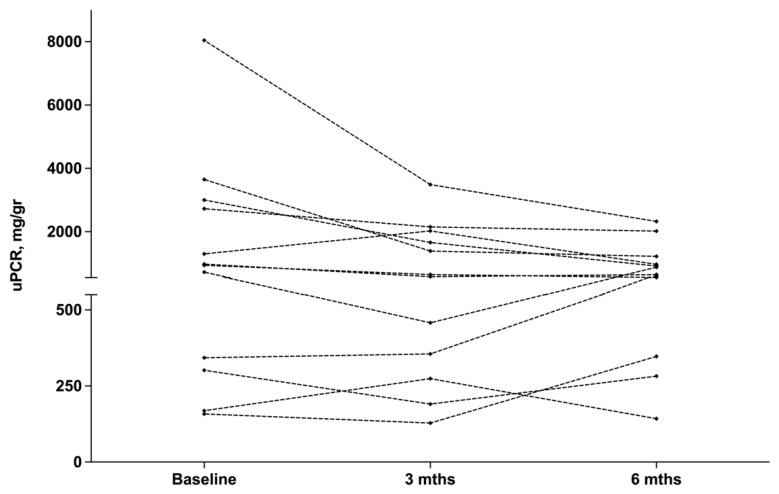
Trend of total proteinuria.

In our cohort of KTRs, a prominent reduction in urinary proteins was observed in the initial phases of therapy, followed by a relative stabilization of the observed effect, but characterized by interindividual variability; the effect appears to be more prominent in patients with higher baseline proteinuria (Figure 2). Despite the overall reduction in 24-h proteinuria and protein-to-creatinine ratio, when analyzed separately, albuminuria did not show statistically significant changes after 3 and 6 months of finerenone therapy, possibly suggesting a more prominent effect on tubule–interstitial rather than glomerular proteinuria in KTRs. In this category of patients, proteinuria frequently comprises a substantial component deriving from tubule–interstitial damage, rather than from glomerular loss [5,6,7,8]. However, the lack of direct measurements of tubular proteinuria limits this hypothesis, underscoring the need for further studies.

Our findings are corroborated by data from the analysis by Kahvecioglu et al. on 15 KTRs, showing a decrease in proteinuria in the patients using finerenone, which was more pronounced in the 1st month, reaching a 40% reduction at the end of the 6-month follow-up, but with no significant difference to the non-randomized control group was found [22].

In the study by Baskin et al., the spot urinary protein-to-creatinine ratio was lower in patients treated with eplerenone than in the control group [26], thus partially agreeing with results from this study and from Kahvecioglu et al. It must be noted that our analysis did not include a control group, and Kahvecioglu et al. did not analyze ratios to urinary creatinine.

Conversely, in the work by Mortensen et al., spironolactone was effective at lowering proteinuria over a 1-year period, but this effect was not sustained in the 2nd and 3rd years; the urinary albumin-to-creatinine ratio was not different between treatment and placebo groups [23].

No relevant variation in blood pressure was observed in our KTR cohort after therapy with finerenone. These data are confirmed in the study by Baskin et al., in which eplerenone had no relevant effects on systolic or diastolic blood pressure in comparison to the placebo group [26]. Similarly, in the retrospective study by de Sousa et al. on 140 KTRs treated with spironolactone, blood pressure remained stable during the 1-year observation period [25].

Conversely, in the work by Mortensen et al., spironolactone was effective at lowering systolic blood pressure, with a statistically significant difference after 1 year of treatment; no differences were found in diastolic blood pressure [23].

A low incidence of adverse effects on serum potassium has been reported in this study; these results align with data from the analysis by Kahvecioglu et al. on 15 KTRs and show no significant difference in serum potassium between the treatment and control groups, despite a significant increase in kalemia in the first month of treatment, followed by a decrease towards baseline levels in the following period. Four patients had clinically important hyperkalemia, leading to discontinuation, but there was no need for hospitalization or dialysis.

Further evidence of low impact on serum electrolytes comes from the work by Mortensen et al., in which spironolactone did actually increase serum potassium in comparison to placebo, but no administration of potassium-lowering drugs was needed [23].

Furthermore, in the study by Baskin et al., eplerenone had no relevant effects on serum potassium in comparison to placebo the group [26]. In the retrospective study by de Sousa et al. on 140 KTRs treated with spironolactone, no relevant effects were observed on serum potassium concentration [25], agreeing with previously mentioned data.

As reported by Morales et al. in their study on 64 KTRs treated with spironolactone or placebo, potassium levels were higher in the treatment group, and 3 patients showed severe hyperkalemia [24], indicating a similarly low rate of side effects.

This study presents some limitations: the small sample size and the lack of a randomized control group limit the robustness of the results, while the relatively short follow-up time cannot evaluate long-term outcomes on graft survival. Furthermore, due to its observational nature, a causative relation cannot be inferred. On the other hand, it is the first work to address the possible benefits and adverse effects of NS-MRSAs in diabetic KTRs already undergoing therapy with renin–angiotensin system inhibitors and SGLT2 inhibitors, addressing urinary protein- and albumin-to-creatinine ratio, thus extending the very limited knowledge currently available. Our results, in conjunction with the findings reported by Kahvecioglu et al., indicate a potential therapeutic role for finerenone in both diabetic and non-diabetic kidney transplant recipients. This could broaden its clinical applicability, analogous to the expanded use observed with sodium–glucose cotransporter-2 inhibitors [27].

## 4. Materials and Methods

This analysis is part of the “Salerno CKD Cohort Study”, an ongoing, open-ended observational study on the whole spectrum of chronic kidney disease, including KTR [28]. The study was reviewed and approved by the local institutional ethical committee (2012—n. 589) and was performed in accordance with the Code of Ethics of the World Medical Association (Declaration of Helsinki). It included written informed consent and was registered in the public registry of the Italian Drug Agency (Agenzia Italiana del FArmaco, AIFA, ID n. 654) and in the public registry of the Authority for Privacy of the Italian Parliament (Garante della Privacy, n. 102400183803). The standard care of KTR patients covered by the Italian National Health Service includes 3–4 outpatient visits per year with lab work-up and hospitalization if needed, regardless of where the transplantation was originally performed.

Inclusion criteria in the analysis were age ≥ 18 years, KTR age ≥ 1 year, eGFR > 25 mL/min/1.73 m^2^, proteinuria while on treatment for at least 12 weeks with renin–angiotensin system inhibitors at the maximum tolerated dose and dapagliflozin 10 mg/day, and presence of diabetes per the requirements of the Italian Drug Agency [29]. Enrollable patients were prescribed 10 mg/day finerenone on top of the ongoing treatment and underwent clinical and lab assessments before the treatment (baseline), after three and six months of treatment. An additional evaluation of serum creatinine and serum electrolytes was performed at 1 month in order to exclude any potential side effects on renal function and serum potassium. In addition to total proteins, albumin, and creatinine in 24-h urine, the data collection included sex, patient age, transplant age at enrollment, body weight, blood pressure in the sitting position, ongoing treatments against hypertension and diabetes, serum creatinine, and serum glucose. Regarding adverse events, hyperkalemia (defined as serum potassium > 5.5 mg/dL) and hypotension (defined as systolic blood pressure < 90 mmHg) were monitored at each visit.

Patients were advised to promptly contact the unit in the event of any new symptom, with special focus on dizziness, asthenia, muscle weakness, numbness, tingling, palpitations, chest pain, and irregular heart rhythms. Automated biochemistry was used for lab measurements in serum and urine.

Urinary proteins were also expressed as a ratio to urinary creatinine. Serum creatinine calibrated with IDMS-traceable standard [30], gender, and age were used for the calculation of the estimated glomerular filtration rate (eGFR) by the CKD-Epi equation [31]. Diabetes was defined as ongoing antidiabetic drug therapy, fasting plasma glucose ≥ 126 mg/dL or glycated hemoglobin (A1C) ≥ 6.5% (≥48 mmol/mol) [32]. Hypertension was defined as ongoing anti-hypertensive therapy or as systolic blood pressure above 130 mmHg and diastolic blood pressure above 80 mmHg [33].

Descriptive statistics were reported as prevalence of categorical variables, mean ± SD of non-skewed numerical variables, and median with inter-quartile range (IQR) of numerical skewed variables (skewness > 1). For repeated measures, a paired t-test was performed for normally distributed variables, while the Wilcoxon signed-rank test was used for skewed variables. Statistical analyses were performed using SPSS 26 (IBM, Armonk, NY, USA).

## 5. Conclusions

In conclusion, despite its small sample size, this study represents the first evaluation of non-steroidal mineralocorticoid receptor antagonists in a cohort of diabetic kidney transplant recipients. In this small cohort of KTRs, finerenone was generally well tolerated, despite a single case of hyperkalemia, and was associated with an early reduction in overall proteinuria and the urinary protein-to-creatinine ratio, while no clinically relevant effects were observed on albuminuria when analyzed separately. No adverse effects on graft function were detected during follow-up. Due to study design, no cause-effect relation between finerenone therapy and reduction in proteinuria can be implied, despite the positive association.

Further studies are needed to expand knowledge and provide more robust evidence before widespread clinical application. The ongoing EFFEKTOR trial (NCT06059664) [34] is a phase 2 randomized, double-blinded, placebo-controlled clinical trial to determine the feasibility, tolerability, safety, and efficacy of finerenone in kidney transplant recipients. Its estimated completion is due in September 2027 and will hopefully add high-quality evidence on this topic.

## Figures and Tables

**Table 1:** Baseline characteristics: mean ± SD for non-skewed data and median (IQR) for skewed data (>1).

Number of patients	13
Men, %	76.9
Age, years	57.7 ± 14
Primary kidney disease	
Unknown, %	38.5
Glomerulopathies, %	30.8
Genetic, %	30.8
Transplant age, years	12.6 ± 7
Body weight, kg	78 ± 14
Diabetes, %	100
Hypertension, %	84.6
Systolic blood pressure, mmHg	145 ± 20
Diastolic blood pressure, mmHg	78 ± 10
Immunosuppressive drugs	
Steroid, %	100
CNi, %	84.6
mTORi, %	61.5
Mycophenolate, %	23.1
Serum glucose, mg/dL	113 ± 23
Glycated hemoglobin, %	6.6 ± 0.9
Serum urate, mg/dL	5 ± 1
Serum creatinine, mg/dL	1.73 ± 0.51
eGFR, mL/min/1.73 m^2^	44 ± 15
Serum sodium, mEq/L	139 ± 2
Serum potassium, mEq/L	4.3 ± 0.4
Serum magnesium, mg/dL	1.94 ± 0.19

eGFR, estimated glomerular filtration rate; CNi, calcineurin inhibitors; mTORi, mammalian target of rapamycin inhibitors.

**Table 2:** Changes in clinical and laboratory variables after 3- and 6-month therapy. Median (IQR) and Wilcoxon test for skewed data; mean ± SD and T-test for non-skewed data.

	Baseline	3 Months	6 Months
Number of Patients	13	12	12
Body weight, kg	78 ± 14	78 ± 14 ^§^	78 ± 16 ^§^
Systolic blood pressure, mmHg	145 ± 20	138 ± 13 ^§^	138 ± 13 ^§^
Diastolic blood pressure, mmHg	78 ± 10	79 ± 10 ^§^	80 ± 10 ^§^
Serum glucose, mg/dL	113 ± 23	113 ± 23 ^§^	105 ± 36 ^§^
Serum urate, mg/dL	5 ± 1	5.4 ± 1.4 ^§^	4.9 ± 0.9 ^§^
Serum creatinine, mg/dL	1.73 ± 0.51	1.85 ± 0.68 ^§^	1.83 ± 0.66 ^§^
eGFR, mL/min/1.73 m^2^	44 ± 15	43 ± 17 ^§^	42 ± 15 ^§^
Serum sodium, mEq/L	139 ± 2	139 ± 1 ^§^	139 ± 3 ^§^
Serum potassium, mEq/L	4.3 ± 0.4	4.4 ± 0.6 ^§^	4.4 ± 0.4 ^§^
Serum magnesium, mg/dL	1.94 ± 0.19	1.90 ± 0.20 ^§^	1.87 ± 0.15 ^§^
Urinary proteins, mg/24 h	1794 (395–3979)	840 (363–2682) *	1011 (417–1908) **
Urinary albumin, mg/L	436 (34–1076)	191 (40–736) ^§^	150 (80–369) ^§^
Urinary protein/creatinine, mg/g	980 (322–2867)	615 (294–1937) ^§^	768 (400–1162) **
Urinary albumin/creatinine, mg/g	715 (83–2045)	315 (85–1507) ^§^	296 (165–604) ^§^

^§^ *p* > 0.05; * *p* = 0.05; ** *p* < 0.05; no statistically significant changes were found between values at 3 vs 6 months.

## Data Availability

The datasets used and analyzed during the current study are not public due to privacy issues as required by the local institutional ethical committee “Campania 2—Ufficio A.O.U. San Giovanni di Dio e Ruggi d’Aragona” (2012 n. 589). Anonymized data will be available from the corresponding author on reasonable request.

## Abbreviations

The following abbreviations are used in this manuscript:

CKD	Chronic Kidney Disease
CNi	Calcineurin Inhibitors
DBP	Diastolic Blood Pressure
eGFR	Estimated Glomerular Filtration Rate
KTR	Kidney Transplant Recipient
mTORi	Mammalian Target of Rapamycin Inhibitors
NS-MRSA	Non-Steroidal Mineralocorticoid Receptor Antagonist
SGLT2i	Sodium–Glucose Cotransporter Type 2 Inhibitors
SBP	Systolic Blood Pressure
